# Deformation Monitoring and Analysis of Baige Landslide (China) Based on the Fusion Monitoring of Multi-Orbit Time-Series InSAR Technology

**DOI:** 10.3390/s24206760

**Published:** 2024-10-21

**Authors:** Kai Ye, Zhe Wang, Ting Wang, Ying Luo, Yiming Chen, Jiaqian Zhang, Jialun Cai

**Affiliations:** 1 College of Environment and Resources, Southwest University of Science & Technology, Mianyang 621010, China; yk17311738297@163.com (K.Y.); ly520xy2023@163.com (Y.L.); 17628285006@163.com (Y.C.); zjq19980407@163.com (J.Z.); caijialun@my.swjtu.edu.cn (J.C.); 2 School of Life Science and Engineering, Southwest University of Science & Technology, Mianyang 621010, China; wangting20030107@163.com

**Keywords:** time-series InSAR, Sentinel-1A, Baige landslide, multi-orbit fusion, deformation analysis

## Abstract

Due to the limitations inherent in SAR satellite imaging modes, utilizing time-series InSAR technology to process single-orbit satellite image data typically only yields one-dimensional deformation information along the LOS direction. This constraint impedes a comprehensive representation of the true surface deformation of landslides. Consequently, in this paper, after the SBAS-InSAR and PS-InSAR processing of the 30-view ascending and 30-view descending orbit images of the Sentinel-1A satellite, based on the imaging geometric relationship of the SAR satellite, we propose a novel computational method of fusing ascending and descending orbital LOS-direction time-series deformation to extract the landslide’s downslope direction deformation of landslides. By applying this method to Baige landslide monitoring and integrating it with an improved tangential angle warning criterion, we classified the landslide’s trailing edge into a high-speed, a uniform-speed, and a low-speed deformation region, with deformation magnitudes of 7~8 cm, 5~7 cm, and 3~4 cm, respectively. A comparative analysis with measured data for landslide deformation monitoring revealed that the average root mean square error between the fused landslide’s downslope direction deformation and the measured data was a mere 3.62 mm. This represents a reduction of 56.9% and 57.5% in the average root mean square error compared to the single ascending and descending orbit LOS-direction time-series deformations, respectively, indicating higher monitoring accuracy. Finally, based on the analysis of landslide deformation and its inducing factors derived from the calculated time-series deformation results, it was determined that the precipitation, lithology of the strata, and ongoing geological activity are significant contributors to the sliding of the Baige land-slide. This method offers more comprehensive and accurate surface deformation information for dynamic landslide monitoring, aiding relevant departments in landslide surveillance and management, and providing technical recommendations for the fusion of multi-orbital satellite LOS-direction deformations to accurately reconstruct the true surface deformation of landslides.

## 1. Introduction

China, with its vast land area and complex geological environment, faces the highest population threat from geological disasters globally. According to the *China Statistical Yearbook*, there have been 241,788 landslide disasters in China since 2000, resulting in direct economic losses of CNY 53.8 billion. Landslides are the most frequent and economically damaging type of geological disaster in the country. Therefore, the protection and management of landslide disasters hold significant practical importance and substantial scientific research value, with management heavily reliant on monitoring. Currently, landslide monitoring technologies are categorized into three main types based on the platforms used and the methods for detecting deformation: ground-based monitoring [1,2], aerial remote sensing monitoring [3,4], and satellite remote sensing monitoring [5,6,7].

**Figure 1:**
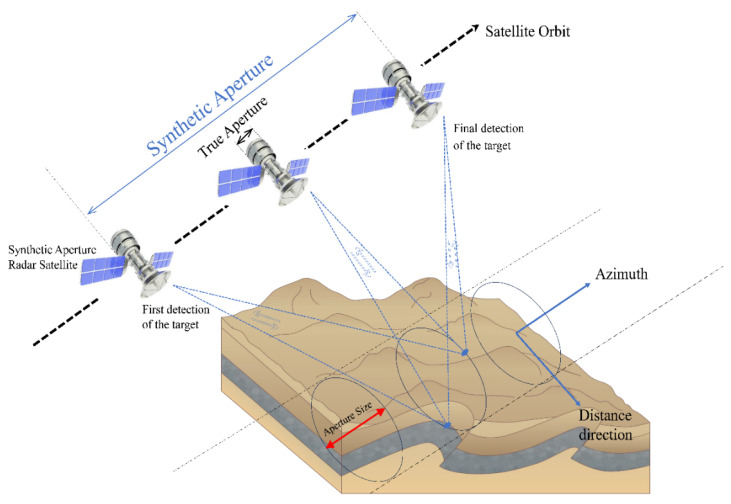
InSAR technology imaging mode.

Ground-based monitoring technology often faces limitations due to environmental terrain and other factors, resulting in only for discrete distributed monitoring. This approach struggles to meet the demand for real-time dynamic monitoring of landslide areas. In contrast, remote sensing technology can overcome these terrain limitations, enabling regular and continuous monitoring of landslide regions in a flexible and mobile manner. Consequently, remote sensing monitoring is gradually supplanting ground-based methods to become the primary means of landslide surveillance. Nevertheless, conventional UAV remote sensing and optical satellite remote sensing remain constrained by meteorological conditions, geometric distortions, image acquisition frequency, and the absence of deformation data due to surface vegetation cover. These limitations prevent the achievement of all-weather landslide monitoring, rendering these methods only appropriate for medium- to long-term deformation trend analysis and hazard assessment in more open areas. Conversely, satellite-borne synthetic aperture radar differential interferometry (InSAR) technology offers extensive image coverage, high spatial and temporal resolution (as shown in Figure 1), and minimal disruption from clouds and surface vegetation. This technology overcomes meteorological and environmental constraints, facilitating dynamic, continuous, all-weather, and all-day monitoring of landslides across entire geomorphological landscapes. Hence, InSAR technology is more adept than traditional remote sensing techniques for monitoring large-scale landslide deformations in the field [8].

The conventional SAR image processing technique can achieve centimeter-level deformation results in the study area following the interferometric phase processing by D-InSAR. However, D-InSAR processing is susceptible to spatial and temporal incoherence, atmospheric disturbances, and geometric distortions, which may compromise the resolution of SAR images and the reliability of deformation results, thereby presenting certain limitations. Consequently, some researchers have introduced a temporal sequence InSAR (time-series InSAR) processing method. This approach involves performing D-InSAR processing on a series of SAR images taken over different periods but covering the same area to extract the necessary surface deformation information. This method is characterized by high monitoring accuracy, resistance to phase error interference, and the ability to determine the deformation pattern of the monitoring target in chronological order. It effectively overcomes the technical limitations of D-InSAR processing, enhancing the accuracy of surface deformation measurements from the centimeter level to the millimeter level [9,10,11].

However, the deformation information derived from InSAR technology is essentially the projection of actual surface deformation onto the satellite’s line-of-sight (LOS) direction. Consequently, significant discrepancies often arise when processing different orbital image data of the same landslide using the same InSAR solution, and substantial deviations are also observed when different InSAR solutions are employed to process the same dataset. Consequently, employing InSAR technology to process single-orbit satellite imagery data often proves challenging in objectively reflecting the comprehensive and authentic surface deformation information of the study area. Hence, one of the pivotal directions for the future development of InSAR technology is undoubtedly how to reconstruct the real surface deformation of landslides by referencing multi-orbit LOS direction deformation data from satellites [12,13,14,15]. This paper focuses on the Baige landslide in China. By utilizing time-series InSAR technology to process 30 scenes each of Sentinel-1A satellite ascending and descending orbit images, we obtained multi-orbit deformation data of the landslide. Based on the geometric principles of satellite line-of-sight imaging, we developed a method to extract landslide’s downslope direction deformation by fusing ascending and descending orbital time-series deformation results. Using this method, we conducted a retrospective analysis of the historical deformation of the Baige landslide and cross-validated the calculated accuracy with actual monitoring data of the landslide, striving to achieve more comprehensive and precise surface deformation information. Finally, the characteristics and source of the landslide deformation were analyzed, and the factors contributing to the landslide activity were explored, by combining the calculated time-series deformation results with the meteorological and geological data around the Baige landslide.

## 2. Study Area and Datasets

### 2.1. Overview of the Study Area

**Figure 2:**
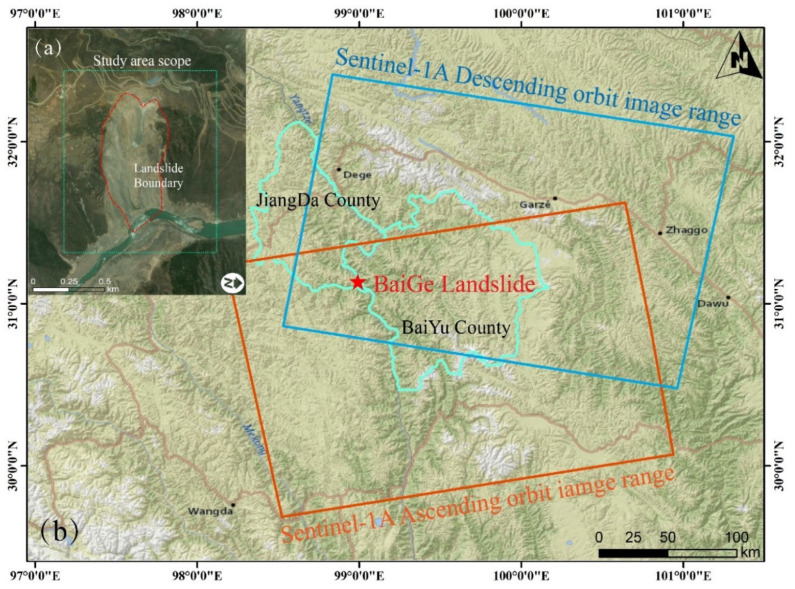
Main body of the landslide and research area, where (**a**) is the main body of the landslide and the extent of the study area and (**b**) is the location of the Baige landslide.

On 11 October and 3 November 2018, two successive, extraordinarily large, high-level rock landslides, collectively known as the Baige landslide, occurred at Baige Village, straddling the border between Jiangda County in Changdu City, Tibet, and Baiyu County in Ganzi Prefecture, Sichuan Province, China; the specific location is shown in Figure 2. These landslides displaced approximately 31.65 million cubic meters of material, obstructing the main flow of the Jinsha River. This obstruction resulted in the formation of a 270 m long, 60 m high barrier, which severed the river’s course and created a dam that inundated the upstream villages and towns of Boluo and Jinsha [16]. Subsequently, the dam’s collapse caused downstream flooding, destroying numerous bridges and inundating several towns, ultimately resulting in direct economic losses estimated at about CNY 6.8 billion. The event posed a grave threat to the safety of the neighboring populace, endangering the lives of many residents in the surrounding areas.

The Baige landslide is situated at the eastern edge of the Tibetan Plateau, characterized by a cold, temperate, semi-humid climate. The landslide area experiences significant temperature variations, with an average annual temperature of 4.5 °C. Over the past five years, the average annual precipitation has been 650 mm, though its spatial and temporal distribution is uneven, with the rainy season occurring predominantly from May to September. The main body of the landslide lies on the right bank of the Jinsha River within a “V”-shaped valley, with a slope orientation of 80° to 100°. The top of the slope reaches an elevation of 3732 m, while the valley floor is at 2882 m, resulting in a height difference of approximately 850 m. The summit of the landslide slope features a gently sloping platform; with a terrace-like terrain at the trailing edge and nearly vertical steep fronts, averaging a gradient of about 60°, the terrain is notably steep [17,18]. Based on existing research and geological data, the upper part of the landslide body primarily consists of serpentinite, situated at an elevation of 3000 m to 3800 m. It generally appears as gray-green fragmented powder rock, characterized by poor tightness in its properties and overall structure, and it easily softens and muddies when exposed to water. Due to long-term weathering, erosion, and tectonic movements, the rock mass becomes fractured, significantly reducing the geological stability of the landslide area [9]. The lower part of the landslide body is mainly composed of dioritic gneiss, with a weathered surface that appears yellow-gray-green. The strata dip at an orientation of 235°∠40°, and two sets of joints have developed at 65°∠56° and 120°∠37° [19]. This indicates that the steep terrain and elevation difference within the landslide area provide favorable potential energy conditions for the occurrence of landslides, while the rock properties and precipitation levels offer the necessary material conditions for their occurrence [20].

After the landslide occurred, field monitoring revealed that the main sliding direction was 105°. The landslide measured 1413 m in length, with an average width of 560 m and an average thickness of 40 m. The total volume of the displaced material was approximately 31.65 million cubic meters, categorizing it as a massive translational landslide [21]. Post-slide, the landslide resembled an armchair, with the main sliding body forming a wedge shape. The cross-sectional profile exhibited alternating steep and gentle steps [22].

### 2.2. Data Sources

This study employs C-band Sentinel-1A satellite imagery as the experimental dataset. Detailed data for the 30-view pre- and post-landslide Sentinel-1A ascending and 30-view descending orbit images are shown in Table 1. The specific orbital parameters for the ascending and descending satellite orbit are provided in Table 2:

## 3. Methodology

### 3.1. Time-Series InSAR Technology

SBAS-InSAR technology [23,24,25,26] utilized a series of images of the study area, captured at the same location over different times, as the data source. The SAR dataset is divided into multiple small baseline subsets, and then phase unwrapping and solving are performed using the least squares method or singular value decomposition. The atmospheric effects are then reduced by a series of low-pass and high-pass filtering in the temporal and spatial domains [27,28], ultimately resulting in high-precision time-series deformation in the LOS direction of the landslide. The critical steps are outlined in Equation (1):

(1) $$\left\{\begin{array}{c} \begin{array}{c} \frac{N+1}{2}\leq M\leq N\left(\frac{N+1}{2}\right)\\ ∆\varphi \left(x,r\right)=\varphi \left(T_{B},x,r\right)-\varphi \left(T_{A},x,r\right)\\ ∆\varphi \left(x,r\right)=\frac{4\pi }{\lambda }\left[d\left(T_{B},x,r\right)-d\left(T_{A},x,r\right)\right]+∆\varphi top\left(x,r\right)+∆\varphi orb+∆\varphi res\left(x,r\right)\\ ∆\varphi \left(x,r\right)=X\left[\varphi final\left(x,y\right)\right]\\ \varphi final\left(x,y\right)={{(X}^{T}X)}^{-1}X^{T}\;∆\varphi \left(x,r\right) \end{array} \end{array}\right.$$

where *M* represents the number of potential differential interferograms, while (*N* + 1) signifies the count of imported images. The symbol λ denotes the radar wavelength, which, for the purposes of this paper, is set to 5.6 cm, corresponding to the C-band satellite imagery. T_A_ and T_B_ indicate the times at which the two SAR images were acquired, and (*x, r*) specifies the position of the SAR image pixel. The term $d\left(T_{B},x,r\right)-d\left(T_{A},x,r\right)$ signifies the deformation accumulated in the line-of-sight (LOS) direction for T_A_ and T_B_ relative to the initial image time T0. The variable $∆\varphi top\left(x,r\right)$ accounts for the phase connection due to DEM error, while ∆φorb represents the computational error stemming from inaccurate satellite orbit data, which is minimal due to the provision of precise orbital data by Sentinel-1. ∆φres(x,r) encompasses all types of errors arising from the residual phase. Finally, *X* is the *M × N* order matrix derived from Equations (2) and (3), and φfinal(x,y) denotes the estimated deformation value obtained.

PS-InSAR technology [29,30] selects the image with the minimum spatial and temporal decorrelation as the master image and registers other images to it. Then, the permanent scatterer (PS) points are extracted, and a functional relationship model is established for differential interferometric phase, DEM error, and deformation phase in both temporal and spatial dimensions using the extracted PS points. Subsequently, the time-series deformation for each PS point is computed. The key steps are illustrated in Equation (2):

(2) $$\left\{\begin{array}{c} \begin{array}{c} D_{x}=\frac{\tau_{x}}{m_{x}}≅\tau_{v}<0.25\\ ∆\varphi_{c,d}\left(P_{x}\right)=∆\varphi_{c,d}^{H}\left(P_{x}\right)+∆\varphi_{c,d}^{DISP}\left(P_{x}\right)+∆\varphi_{c,d}^{A}+∆\rho_{c,d}\left(P_{x}\right)\\ \varepsilon \left[∆v\left(Pxy\right),∆h\left(Pxy\right)\right]=\frac{1}{N}\sum_{i=1}^{N}e^{y\left[∆\varphi_{ij}\left(P_{xy}\right)-\frac{4\pi }{\lambda }∆v\left(P_{xy}\right)B_{ti}-\frac{4\pi }{\lambda R\sin\theta }∆h\left(P_{xy}\right)B_{ni}\right]}\\ \left[∆\tilde{v}\left(P_{xy}\right),∆\tilde{h}\left(P_{xy}\right)\right]=argmax\left\{\left|\varepsilon \left[∆v\left(Pxy\right),∆h\left(Pxy\right)\right]\right|\right\}\\ \tilde{\varepsilon }\left(P_{xy}\right)=max\left|\varepsilon \left[∆\tilde{v}\left(P_{xy}\right),∆\tilde{h}\left(P_{xy}\right)\right]\right| \end{array} \end{array}\right.$$

where $\tau_{x}$ represents the standard deviation of the amplitude, and $m_{x}$ denotes the mean amplitude. $P_{x}$ indicates the position of the XTH permanent scatterer point of the candidate, c and d refer to the interferometric image pairs. $∆\varphi_{c,d}^{H}\left(P_{x}\right)$ signifies the global DEM error induced by the reference DEM error, while $∆\varphi_{c,d}^{DISP}\left(P_{x}\right)$ represents the linear deformation rate. The phase delay due to atmospheric factors is denoted by $∆\varphi_{c,d}^{A}$, and $∆\rho_{c,d}(P_{x})$ reflects the temporal and spatial incoherence values. Pxy denotes the connection between neighboring PSC points Px and Py. N is the number of generated interferograms, *λ* represents the radar wavelength, *θ* indicates the angle of incidence, *R* is the distance between the object of study and the sensor, $∆\varphi_{ij}\left(P_{xy}\right)$ is the bi-differential interferometric phase concerning x and y, $B_{ti}$ is the temporal baseline, and $B_{ni}$ is the interferometric baseline.

### 3.2. Computational Method for Extracting Landslide’s Downslope Direction Deformation by Fusing Multi-Orbit LOS Direction Deformation

**Figure 3:**
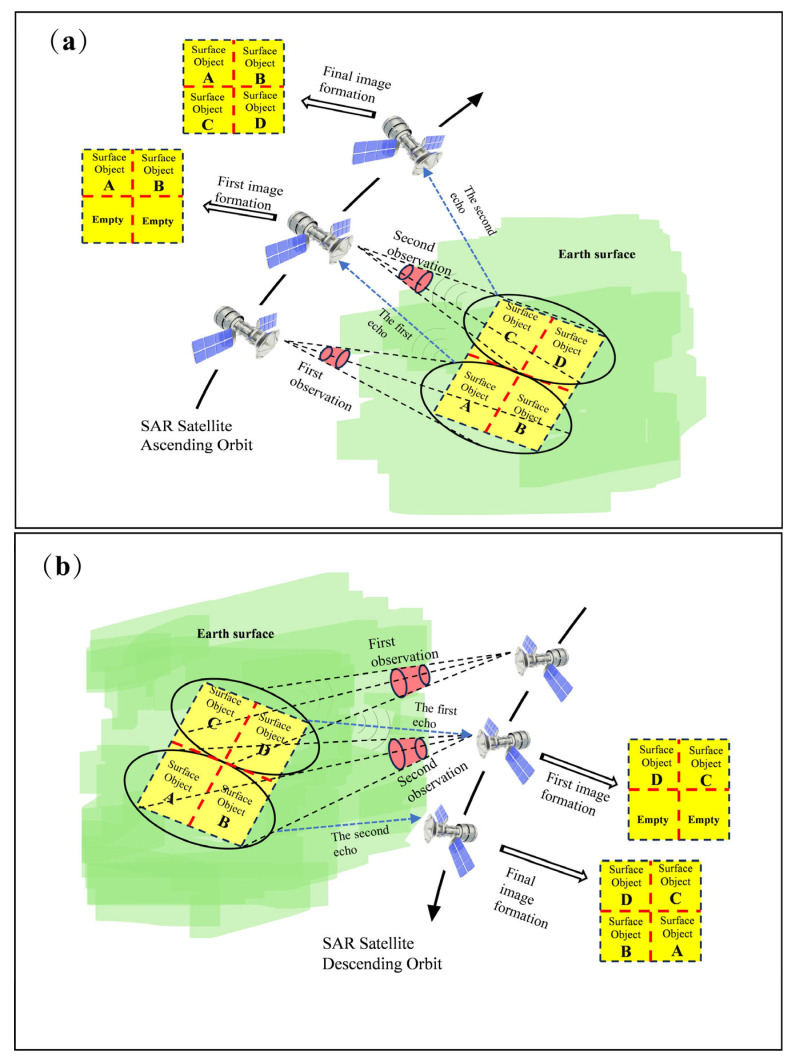
The specific imaging modes of satellites in ascending and descending orbits. Where (**a**) is the ascending orbit satellite imaging mode and (**b**) is the descending orbit satellite imaging mode.

**Figure 4:**
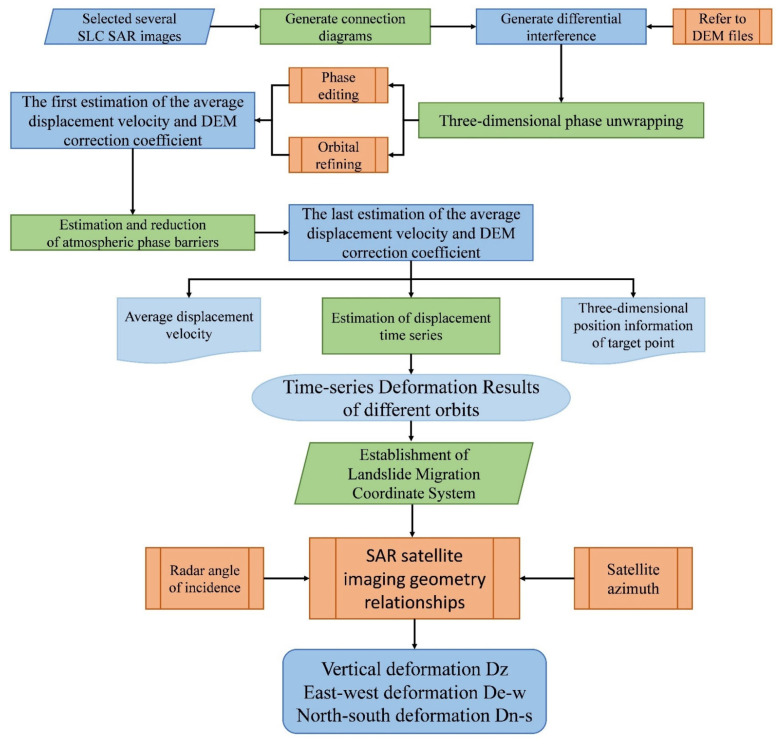
Fusion extraction flow chart.

**Figure 5:**
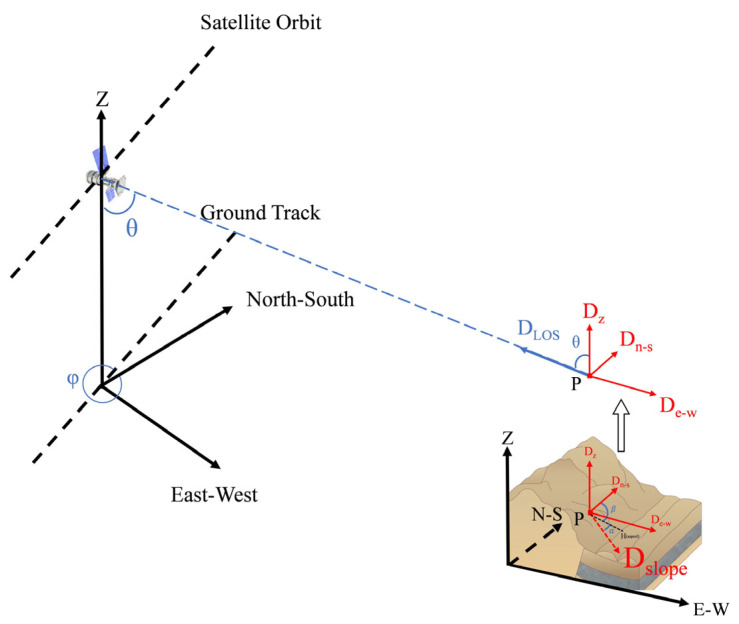
LOS direction deformation transformation model.

This paper employs the SBAS-InSAR and PS-InSAR techniques, using 30 ascending and 30 descending orbit images from the Sentinel-1A satellite as experimental data to calculate fundamental ground deformation information of landslides. The descending orbit indicates the satellite’s movement from the Earth’s North Pole towards the South Pole, while the ascending orbit represents movement from the South Pole towards the North Pole. In different operating orbits, the sequence in which a satellite captures two adjacent images in the along-orbit direction varies. In the north–south direction, an ascending satellite is perceived as moving from the bottom to the top of its orbit. The satellite prioritizes capturing and recording features below the along-orbit direction first, followed by those above. Consequently, the information of features below the along-orbit direction is recorded at the top of the image, while the information of features above the along-orbit direction is recorded at the bottom. This results in the recorded features in the output image of the ascending satellite being vertically inverted compared to the actual features. In the east–west direction, SAR satellites prioritize recording features closer to the satellite in the oblique direction. When capturing images from the right-side view, features on the left are recorded first, followed by those on the right. Therefore, the feature information recorded in images taken by the ascending satellite is vertically inverted relative to the actual features in the study area. Conversely, when the descending satellite captures images from the right-side view, the information recorded in the image is inverted to the left and right with the actual features. The specific imaging modes of satellites in ascending and descending orbits are illustrated in Figure 3 below:

Therefore, the deformation values recorded and output by SAR satellites represent the changes in the radar LOS direction, rather than the true surface deformation values [31,32]. When the satellite is in its descending orbit, if a surface feature subsides, its distance from the satellite in the LOS direction increases, resulting in a positive deformation value for the pixel point where the feature is located. Conversely, if the surface feature rises, its distance from the satellite decreases, yielding a negative deformation value. In ascending orbit, the satellite imaging is vertically inverted relative to the descending orbit image, and the calculated LOS direction deformation results are also reversed, with a negative value indicating subsidence and a positive value indicating uplift. Therefore, it is challenging to directly fuse the LOS direction deformation values obtained from both ascending and descending orbit datasets. To address this issue, it is necessary to decompose the line-of-sight deformation values using the geometric relationship of SAR satellite imaging before merging them. The experimental process is illustrated in Figure 4 below:

First, we established a landslide migration coordinate system for each target point on the landslide surface by integrating the three-dimensional cartesian coordinate system with the synthetic aperture radar Doppler coordinate system, as shown in Figure 5. This categorization enabled us to broadly classify the landslide’s LOS direction deformation into vertical deformation (D_Z_), east–west deformation (D_e-w_), and north–south deformation (D_n-s_).

Given that the Sentinel-1A satellite follows a near-polar orbit, its imaging is relatively insensitive to the north–south component *D_n_*_−_*_s_*. Consequently, the north–south deformation *D_n_*_−_*_s_* can be disregarded during the decomposition of LOS direction deformation. Utilizing the geometric relationship of SAR satellite imaging, coupled with the differences in radar incidence and azimuthal angles between the two orbits [33,34], the two sets of LOS direction deformations can be transformed into vertical deformation *D_Z_* and east–west deformation *D_e_*_−_*_w_* according to Equation (3):

(3) $$\left\{\begin{array}{c} {-D}_{LOS}^{A}=D_{Z}\cos\theta_{inc}^{A}-D_{e-w}\sin\theta_{inc}^{A}\sin\left(\alpha_{azi}^{A}-\frac{3}{2}\pi \right)\\ D_{LOS}^{D}=D_{Z}\cos\theta_{inc}^{D}-D_{e-w}\sin\theta_{inc}^{D}\sin\left(\alpha_{azi}^{D}-\frac{3}{2}\pi \right) \end{array}\right.$$

where $D_{LOS}^{A}$ and $D_{LOS}^{D}$ represent the LOS direction deformations derived from the ascending and descending orbit datasets, $\theta_{inc}^{A}$ and $\theta_{inc}^{D}$ denote the incidence angles of the ascending and descending orbits, $\alpha_{azi}^{A}$ and $\alpha_{azi}^{D}$ correspond to the azimuthal angles of the ascending and descending orbits, respectively. $D_{Z}$ and $D_{e-w}$ indicate the vertical and east–west deformations of the target point, respectively.

Meanwhile, based on the characteristics of the landslide translational movement mechanism, it is assumed that surface features on the landslide only move downward along the slope direction [35,36]. To extract the deformation along the downslope direction of the landslide, further projection and conversion of the vertical deformation component *D_Z_* and the east–west deformation component *D_e__−__w_* are required. To achieve this, one must reference the landslide’s slope and aspect alongside the satellite’s detailed orbital parameters. Among them, the slope and aspect of the landslide have been determined by the landslide itself, but the satellite provides two sets of orbital parameters: those of the ascending and descending orbits. For ascending satellites, images are inverted north to south; for descending satellites, images are inverted east to west. Notably, the Sentinel-1A satellite operates on a near-polar orbit, and its imaging method is not sensitive to the north–south direction deformation of landslides. Therefore, selecting the orbital parameters of ascending satellites as a reference can minimize the impact of the projection process on the vertical component of deformation *D_Z_* and the east–west component *D_e__−__w_*. Furthermore, since the Baige landslide itself moves from east to west, while the descending satellite image is inverted east to west, utilizing descending satellite parameters may have a certain impact on the objectivity of the deformation projection. Therefore, by referring to the orbital parameters of the satellite’s ascending orbit and the geometric relationship of the landslide migration coordinate system, the sum of the projections of the vertical component of deformation *D_Z_* and the east–west component *D_e__−__w_* can be converted into the deformation along the landslide’s downslope direction [37,38,39], Ds, as shown in Equation (4):

(4) $$D_{Slope}=\frac{D_{Z}\cos\theta_{inc}^{A}-D_{e-w}\cos\varphi^{A}\sin\theta_{inc}^{A}}{\cos\varphi^{A}\sin\theta_{inc}^{A}\cos\alpha \sin\beta +\sin\varphi^{A}\sin\theta_{inc}^{A}\cos\alpha \cos\beta -\cos\theta_{inc}^{A}\sin\alpha }$$

where $\theta_{inc}^{A}$ and $\varphi^{A}$ are the incidence angles and heading angle of the ascending orbit satellite, respectively. α is the average slope of the back edge of Baige landslide, that is 75°; β is the average aspect of Baige landslide, that is 92°. $D_{Slope}$ is the deformation of the landslide surface features along the landslide downslope direction.

### 3.3. Landslide Sliding Stage Classification Method

Upon acquiring the landslide time-series deformation results, we applied the improved tangential angle warning criterion [40,41] to classify the deformation stages of the Baig landslide in accordance with the three laws of landslide creep deformation [42,43]. Specifically, when the tangential angle, defined as the size of the tangent line of the displacement–time curve relative to the horizontal axis, ranges from 0 to 45°, it indicates the low-speed deformation stage. Angles between 45° and 60° signify the uniform-speed deformation stage, while angles between 60° and 80° represent the high-speed deformation stage. When the tangential angle approaches 90°, the landslide is in a critical sliding state. In this study, we utilize the median of the average displacement rate, derived from secondary inversion calculations during the time-series InSAR processing, as the base value. This allows for us to recalibrate the vertical axis value (deformation variable) in the displacement–time coordinate system, thus standardizing the scale of the tangential angle warning criterion, as illustrated in Equations (5) and (6):

(5) $${Exc}_{i}=\frac{D_{i}}{V_{Inversion}}$$

(6) $$\alpha_{i}=arctan\frac{{Exc}_{i}-{Exc}_{j}}{∆t}$$

where $D_{i}$ represents the cumulative deformation variable and $V_{Inversion}$ denotes the reference value for the selected displacement rate, with this study opting for the median value of 23 mm/year derived from the secondary inversion calculation of SBAS–InSAR average displacement rate. Δ*t* signifies the monitoring period, and $\alpha_{i}$ is the resulting tangential angle.

## 4. Results

### 4.1. Time–Series InSAR Processing Results

**Figure 6:**
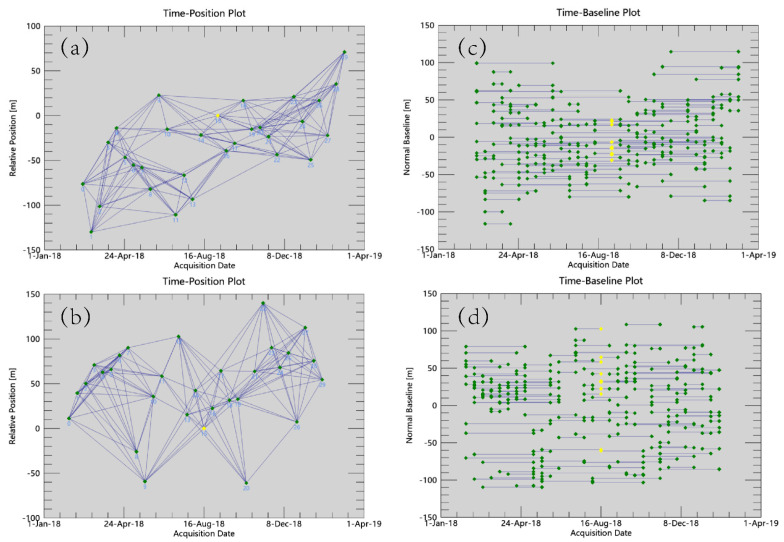
Spatial and temporal baseline maps, where (**a**,**b**) are time–position plots of the descending and ascending orbit images; (**c**,**d**) are time–baseline plots of the descending and ascending orbit images. (These diagrams were drawn using ENVI-SARscape5.6.2 software).

The SBAS–InSAR technique was utilized to process 30 ascending orbit and 30 descending orbit Sentinel-1A satellite images, and the spatial and temporal baseline maps of the two image datasets were obtained as shown in Figure 6 below. The results indicate that both image datasets exhibit excellent spatiotemporal coherence.

**Figure 7:**
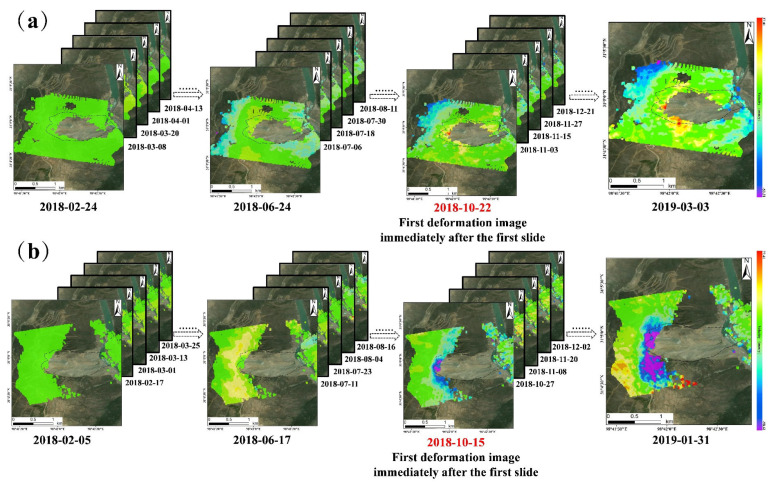
Time-series deformation results obtained from the SBAS-InSAR. Where (**a**) is for the descending orbit dataset and (**b**) is for the ascending orbit dataset.

And the time-series deformation results obtained from the SBAS–InSAR technique are shown in Figure 7 below. The time-series deformation results from the descending orbit dataset comprehensively covered the main body of the landslide and its surrounding area. Notably, there were three distinct deformation areas at the landslide’s trailing edge, and the characteristics are basically consistent with existing studies [44,45,46]. However, the central region of the landslide exhibited significant spatial and temporal incoherence in images due to excessive deformation magnitude, resulting in extensive blank areas. These blank areas are regarded as the primary regions of the landslides. In the time series deformation results from the ascending orbit dataset, the deformation of the landslide’s trailing edge was prominently visible. These deformation areas significantly overlap with the deformation results from the descending orbit dataset, providing a solid foundation for fusion calculations. However, the central region of the landslide displayed extensive blank areas due to spatial and temporal incoherence in the images, rendering further fusion calculations unfeasible.

**Figure A1:**
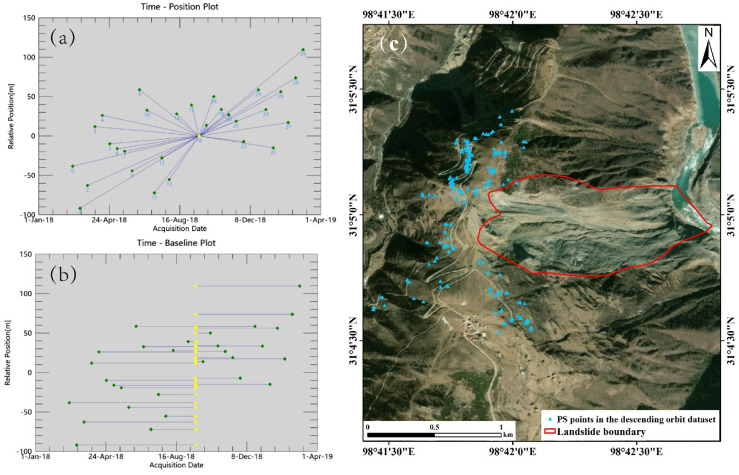
Results from PS-InSAR processing of descending orbit images: (**a**,**b**) are the time-position plot and time-baseline plot of the descending orbit images; (**c**) is a permanent scatterer point position map of the descending orbit images.

**Figure A2:**
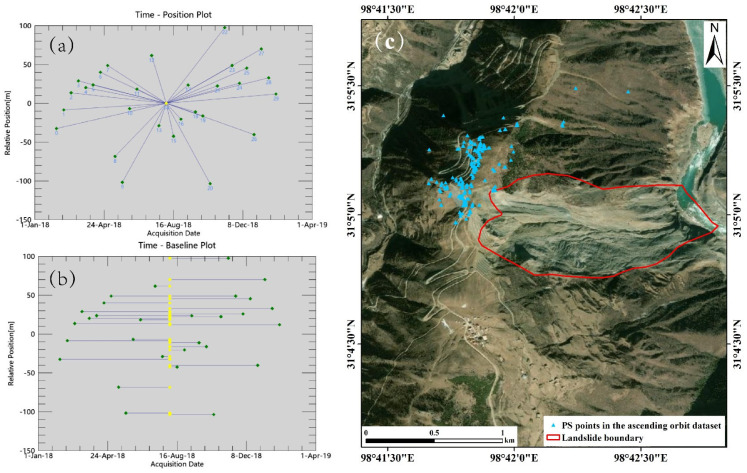
Results from PS-InSAR processing of ascending orbit images: (**a**,**b**) are the time-position plot and time-baseline plot of the ascending orbit images; (**c**) is a permanent scatterer point position map of the ascending orbit images.

However, due to the study area’s mountainous terrain, dense vegetation, and sparse buildings, there are very few permanent scatterer points available for reference. Consequently, the time-series deformation results obtained from the PS–InSAR technique cover only a minimal portion of the landslide’s trailing edge. This limitation rendered the PS–InSAR deformation results insufficient as the primary basis for dynamic deformation analysis and landslide phase delineation. (Refer to Figure A1 and Figure A2 in the Appendix A for the PS–InSAR processing results.)

### 4.2. Landslide’s Downslope Direction Deformations Extracted by Fusion of Multi-Orbit Time-Series Deformations

**Figure 8:**
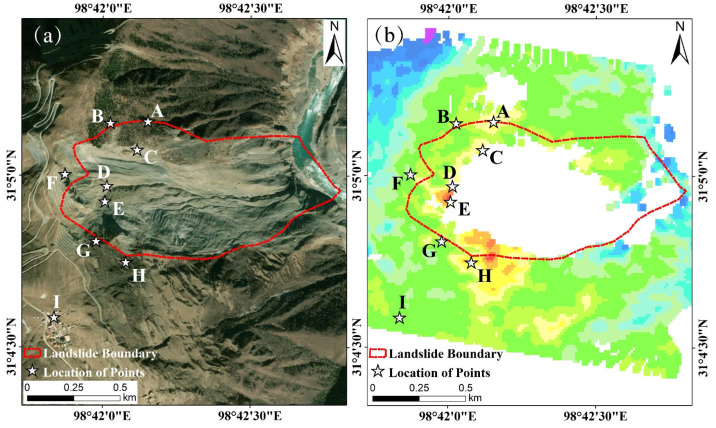
Location of feature points on the trailing edge of the Baige landslide: (**a**) is the location of the points in the satellite map and (**b**) is the location of the points in the descending orbit time-series deformation result.

Due to the substantial deformation magnitude of the Baige landslide’s main body, which exceeds the monitoring capacity of InSAR technology, the central region of the landslide has become significantly incoherent [44]. As a result, the time-series deformation data for this central region appeared blank, rendering it impossible to extract public coherent points. Therefore, in this paper, nine points A~I on the trailing edge of the landslide were selected as feature points in the overlap area of the ascending and descending orbit time series deformation results. The coordinates of each feature point corresponded precisely between the ascending and descending orbit image datasets. Since the descending orbit time series deformation results have higher coverage of the study area, the locations of the feature points were illustrated on the descending orbit time series deformation results map, with specific point locations detailed in Figure 8 below. Notably, point I is a characteristic point on the road in Baige village, and monitoring its deformation holds considerable practical significance.

**Figure 9:**
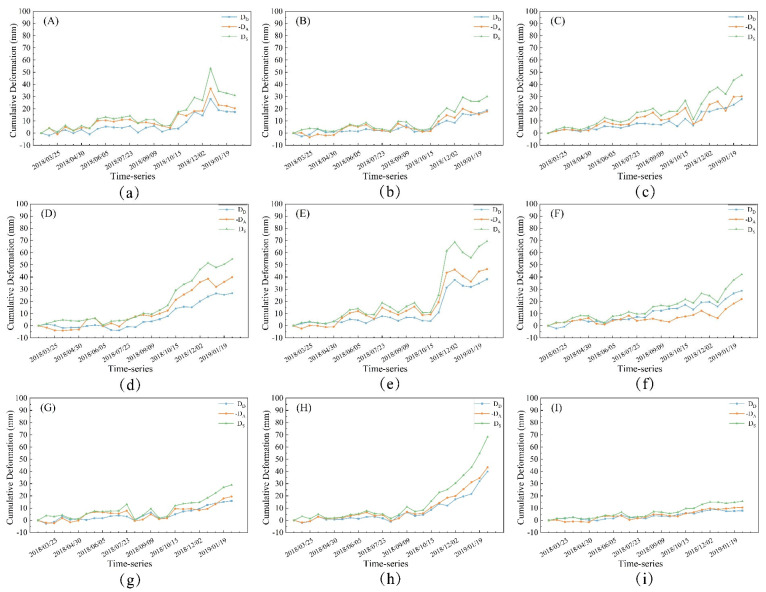
Time-series deformation results for each monitoring point. Where (**a**–**i**) are the time-series deformations of the corresponding monitoring points (A–I). The blue line is the descending orbit LOS direction time-series deformation, the orange line is the ascending orbit LOS direction time-series deformation, and the green line is the landslide’s downslope direction time-series deformation.

The computed landslide’s downslope direction deformations (D_S_), descending orbit LOS direction deformations (D_D_), and ascending orbit LOS direction deformations (-D_A_) at each point are shown in Figure 9 below.

Figure 9 illustrates that the deformation trends in the LOS direction for both ascending and descending orbits are largely consistent at each point. The downslope direction deformation of the landslide, determined through data fusion, also aligns well with these trends. Following the landslide slip, the deformations at points A, B, and C, located on the right side of the landslide’s trailing edge, exhibited significant fluctuations before gradually stabilizing and showed tangential angles of 42.4°, 33.2°, and 53.8°, respectively, indicating a stage of low-speed and uniform-speed deformation. In contrast, the deformations at points D through F, situated in the middle of the landslide’s trailing edge, increased markedly post-slip and continued to accelerate, with tangential angles of 64.3°, 70.5°, and 60.1°, respectively, indicating a high-speed deformation stage. The deformations at points G and H on the left side of the trailing edge exhibited a steady growth trend, with tangential angles of 42.6° and 76.9°, respectively. Point G was in a stage of uniform-speed deformation, whereas point H was in a high-speed deformation stage. Near Baige Village, the deformation at point I remained more stable, with a tangential angle of 24.5°, indicative of a low-speed deformation stage.

**Figure 10:**
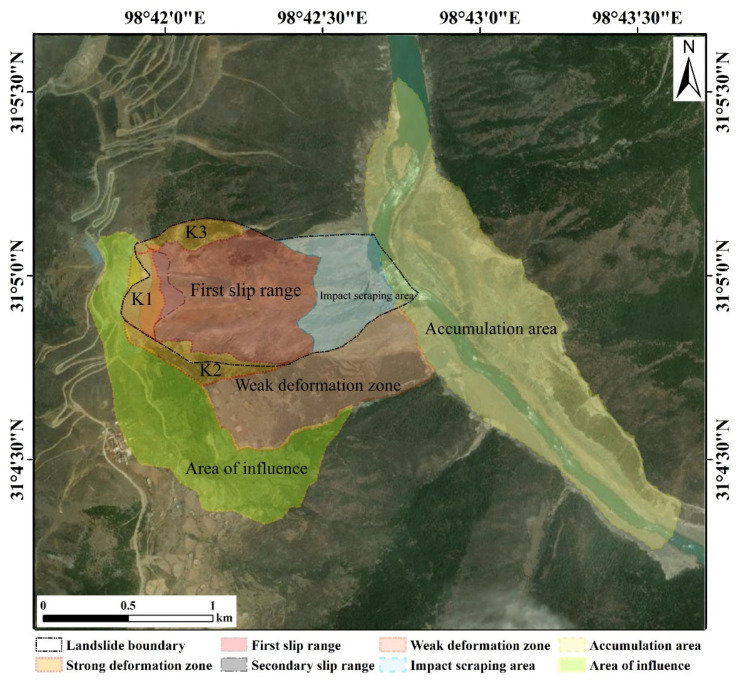
Delineation of landslide areas.

The analysis indicated that the central region of the trailing edge of the landslide, particularly near points D to F, is a strong deformation area. This area, located close to the main body of the landslide, showed a deformation magnitude of about 7~8 cm. Additionally, there are secondary deformation areas on both the left and right sides of the trailing edge. The deformation area on the right side tended to be stabilized, with a deformation magnitude of about 3~4 cm. Conversely, the deformation area on the left side continued to accelerate, with deformation magnitudes between 5 and 7 cm. The specific division of the landslide area is illustrated in Figure 10 below. The results show that the landslide area and sliding stage delineated by the landslide’s downslope direction deformation calculated in this paper were basically consistent with the results obtained by other researchers [22,37,45].

## 5. Discussion

### 5.1. Accuracy Verification of the Fusion Method for Extracting the Landslide’s Downslope Direction Deformation Ds

**Figure 11:**
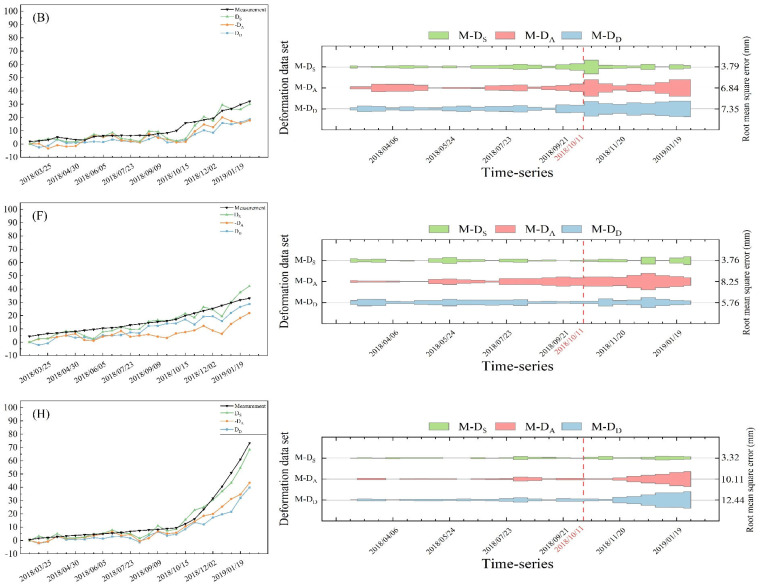
Distribution of errors between each time-series deformation result dataset and measured data, where (B), (F) and (H) represent the deformation information of points B, F and H respectively, M-D_S_ in green is the error between the measured data and the extracted landslide’s downslope direction deformation, M-D_A_ in red is the error between the measured data and the ascending time-series deformation, M-D_D_ in blue is the error between the measured data and the descending time-series deformation, and the red dashed line is the time when the first landslide occurred.

By comparing and analyzing our results with existing studies on the Baige landslide, we determined that the landslide’s downslope direction time-series deformations extracted in this paper exhibited a high degree of consistency with other studies [17,22,44,45], thus confirming the validity and accuracy of our method. Nevertheless, discrepancies still existed among the three sets of time-series deformation results. To address this, we referenced existing measured landslide’s downslope direction deformation data from Baige landslide deformation monitoring [37] and selected three feature points with measured data on the left, middle, and right sides of the landslide’s trailing edge, specifically points B, F, and H. We then calculated the absolute error and root mean square error (RMSE) between each set of time-series deformation data and the measured data at these points. This approach allowed for us to quantify and analyze the deformation error at each point and assess the calculation accuracy. The error distribution between the calculated time-series deformation of each point and the measured data is illustrated in Figure 11:

Figure 11 clearly demonstrated that the errors between the time-series deformation and the measured data for each point basically showed an approximately distribution prior to the landslide slip. However, after the landslide slip, the errors at each point increased significantly. Additionally, larger deformation variables correspond to greater error fluctuations in the feature points. On the whole, the maximum RMSE between the landslide’s downslope direction deformation obtained from fusion extraction and the measured data was only 3.79 mm, which indicates that the method has a high computational accuracy. A comprehensive analysis of the errors at the three points showed that the average RMSE between the fusion extracted landslide’s downslope direction deformation and the measured data was 3.62 mm. In comparison, the average RMSE between the ascending and descending LOS direction deformation and the measured data was 8.40 mm and 8.52 mm, respectively. Compared to the single ascending and descending orbit LOS direction deformation, the RMSE between the fused landslide’s downslope direction deformation and the measured landslide deformation was reduced by 56.9% and 57.5%, respectively. This indicated that the accuracy of the landslide’s downslope direction deformation extracted from the fusion of ascending and descending orbit time-series deformation is significantly improved over that obtained from single orbit SBAS-InSAR processing.

However, this method also has some limitations. Firstly, the landslide’s downslope direction deformation extracted by this method is based on the LOS direction deformation obtained using time-series InSAR technology, and it can only calculate the landslide’s downslope direction deformation in the overlapped area of the multi-orbit time-series deformation results. Consequently, the coverage and calculation accuracy of the extracted landslide’s downslope direction deformation are largely influenced by the deformation results obtained from the time-series InSAR technology. Secondly, existing studies have shown that SAR satellites are most sensitive to vertical deformation, followed by east–west deformation, and are least sensitive to north–south deformation [47]. Therefore, the method demonstrates high sensitivity when the landslide slip direction is east–west but has lower sensitivity when the slip direction is north–south.

### 5.2. Deformation and Causative Factors Analysis of the Baige Landslide

**Figure 12:**
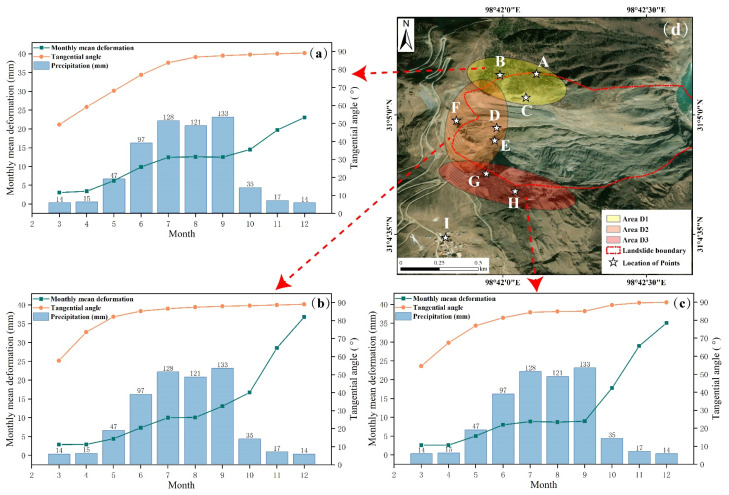
Relationship between tangential angle and monthly mean deformation and rainfall in different regions around the landslide trailing edge. Where (**a**–**c**) are the specific information of Area D1, D2, and D3, respectively, and (**d**) is the location map of the areas.

We analyzed the deformation characteristics and sources of the landslide and explored the factors contributing to the landslide activity by combining the calculated time-series deformation results with meteorological and geological data around the Baige landslide. Based on the distribution of nine selected feature points (A~I), the landslide trailing edge was categorized into three deformation areas: Area D1 on the left (points A~C), Area D2 in the middle (points D~F), and Area D3 on the right (points G and H); these are illustrated in Figure 12d. The monthly average deformation of all feature points in each region is calculated as the monthly deformation of that region, and the tangential angle of each region is calculated according to Equations (5) and (6). The change in deformation and tangential angle in each region in the time series is shown in Figure 12a–c.

In Figure 12, a monthly increasing trend in deformation is evident across the three observed areas. Specifically, in March and April, area D1 exhibits relatively stable deformation due to minimal precipitation, with tangential angles measuring 49° and 54°, indicative of a uniform-speed deformation stage. As precipitation rises in May, deformation in area D1 intensifies, with the tangential angle escalating to 68°, marking the transition to a high-speed deformation stage. In June, when average monthly precipitation approached 100 mm, deformation in area D1 accelerates further, and the tangential angle reaches 77°, approaching the landslide’s critical sliding stage. Under the influence of heavy precipitation from July to September, the tangent angle of the landslide exceeded 85°, and it was in a critical sliding state. It was not until the first landslide occurred in October that the deformation in area D1 increased significantly. Notably, deformation and tangential angle trends in areas D2 and D3 mirror those of area D1. Area D3 enters the landslide’s critical sliding state in July, whereas area D2 does so as early as May. It is inferred that the amount of precipitation may be an important factor causing the landslide to slide. Accordingly, the Pearson correlation coefficient [48,49] between landslide deformation and precipitation was computed using Equation (A1) (see Equation (A1) in Appendix B).

**Figure A3:**
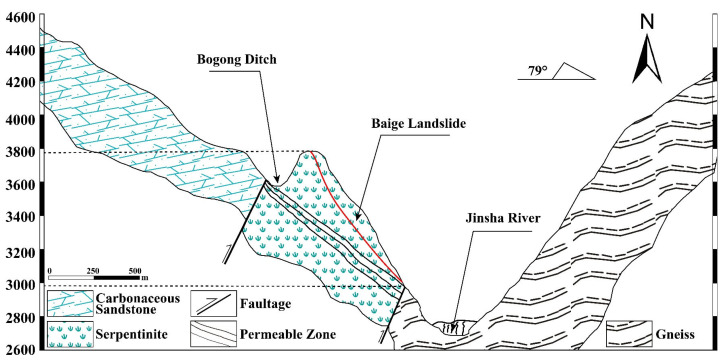
Stratigraphic lithology and topographic map of the Baige landslide.

Landslides exert a significant influence on the deformation of the adjacent trailing edge area during their occurrence. Hence, to compute the correlation coefficient between deformation and precipitation, we solely consider data preceding the first landslide, specifically from March to September. Our calculations reveal strong positive correlations between precipitation and deformation in areas D1, D2, and D3, with correlation coefficients of 0.996, 0.943, and 0.91, respectively, indicating that precipitation is an important factor causing early landslide activity. Upon analyzing deformation patterns after the landslide, it is evident that area D1 was less impacted compared to D2 and D3. Area D2, in particular, exhibited the most significant deformation increase. This indicates that while precipitation predominantly affects area D1, the actual landslide event has a greater influence on D2 and D3. Furthermore, geological investigations and existing research indicate that the upper portion of the landslide consists primarily of serpentinite, a rock type noted for its poor physical properties and structural integrity. When exposed to water, this rock tends to soften and becomes cloudy, significantly contributing to the progression of the Baige landslide [9]. Additionally, the landslide’s vertical drop of approximately 850 m provides ample potential energy for its development [19,20]. These factors together contributed to the early development of the Baige landslide. In addition, China Earthquake Networks data show that there were two earthquakes with magnitudes above 3.0 in July and August in Changdu City, where the Baige landslide is located, and one earthquake with a magnitude above 3.0 in Ganzi Prefecture, which is adjacent to Changdu City, in May and June. At the same time, there were also two earthquakes in Aba Prefecture, which is adjacent to the northeast of Changdu City, and one in Yushu City, which is also northeast of Changdu City, in August. It is speculated that the continuous and intense earthquakes in the surrounding area of the Baige landslide are also one of the factors causing the development of the landslide (For detailed stratigraphic lithology and topographic map, refer to Appendix B, Figure A3).

The analysis indicates that precipitation is a key factor contributing to the Baige landslide. Additionally, the distinct geological environment and stratigraphic composition of the area create favorable potential energy conditions and a material basis for landslide occurrence. Furthermore, the continued and dense earthquakes in the region also play a significant role in the development of the landslide. Furthermore, the primary locations and trends of deformation development in the D1 to D3 regions exhibit a high degree of consistency with the findings of other established studies [18,22,37,46,50].

## 6. Conclusions

Owing to the inherent limitations of the synthetic aperture radar (SAR) satellite imaging method, conventional time-series InSAR technology is capable of recording only one-dimensional deformation in the line-of-sight direction of the landslide. This limitation restricts its ability to fully represent the true deformation information of the landslide surface. However, for the same SAR satellite, the geometric relationship of its imaging in different orbits remains consistent. Therefore, based on the time-series InSAR technology and the imaging geometry of SAR satellites, this paper proposes a computational method to fuse the LOS direction deformation of SAR satellites in multi-orbits to extract the real surface deformation of landslides. This approach transforms the one-dimensional LOS direction deformation captured by SAR satellites into the downslope direction surface deformation of landslides, providing more comprehensive and accurate information on landslide surface deformation.

After applying this method to the monitoring of the Baige landslide in China, we successfully extracted the landslide’s downslope direction deformation of the landslide by fusing the multi-orbit line-of-sight direction time-series deformation from the Sentinel-1A satellite. Meanwhile, we identified three major deformation regions on the trailing edge of the landslide based on the extracted landslide’s downslope direction deformation and the improved tangential angle warning criteria. Among them, there is a high-speed deformation area to the left of the center of the landslide trailing edge, with a deformation magnitude of 7–8 cm, and there is a secondary deformation area on each side of the landslide trailing edge, with a deformation magnitude of 5–7 cm on the left side, which is still accelerating, and 3–4 cm on the right side, which has already stabilized. The monitoring results are highly consistent with existing studies, proving the accuracy and applicability of the method.

Simultaneously, compared to time-series deformation results from a single orbit, the landslide’s downslope direction deformation obtained through the fusion extraction demonstrates significantly enhanced monitoring accuracy. The average root mean square error (RMSE) between the fused landslide’s downslope direction deformation and the measured deformation data of landslide is only 3.62 mm. This was 56.9% and 57.5% less than the average root mean square error between the LOS direction deformation of ascending and descending orbit and the measured data, respectively. It can be seen that the method of extracting landslide’s downslope direction deformations by fusing multi-orbit line-of-sight (LOS) direction deformations can effectively improve the monitoring accuracy of the conventional time-series InSAR technology. We examined the deformation characteristics and origins of the Baige landslide by integrating the calculated time-series deformation results with local meteorological and geological data. The analysis reveals that heavy, concentrated precipitation is an important factor contributing to the activity of the Baige landslide. Additionally, the area’s unique geological environment and stratigraphic composition create favorable conditions for landslide occurrence. Continuous and dense earthquakes in the region further contribute to the landslide’s development. The landslide’s downslope direction deformation extracted based on this method provides a more accurate and comprehensive depiction of landslide deformation information. This, in turn, aids relevant authorities in the continuous monitoring and management of landslides and offers foundational data for predicting future landslide deformation trends, showcasing its considerable application value.

## Figures and Tables

**Table 1:** SAR data sources.

SAR Data Source	Image Time Interval	Scene Heading	Wave Length/cm	Regression Cycle	Image Quantity
Sentinel-1A (Ascending Orbit)	5 February 2018 ~ 31 January 2019	347.28°	5.6 (C Wave band)	12 Days	30 m
Sentinel-1A (Descending Orbit)	24 February 2018 ~ 3 March 2019	192.84°	5.6 (C Wave band)	12 Days	30 m

**Table 2:** Detailed orbit parameter of ascending and descending orbit.

Satellite Orbit	Incidence Angle	Scene Heading	Azimuth Angle
Ascending Orbit	34.439°	347.28°	12.72°
Descending Orbit	34.428°	192.84°	167.16°

## Data Availability

The products presented in this article are available on request from the corresponding author.

## Appendix A

Due to the study area’s mountainous terrain, dense vegetation, and sparse buildings, there are very few permanent scatterer points available for reference. Consequently, the time-series deformation results obtained from the PS-InSAR technique cover only a minimal portion of the landslide’s trailing edge. This limitation renders the PS-InSAR deformation results insufficient as the primary basis for dynamic deformation analysis and landslide phase delineation. The results obtained from PS-InSAR processing are shown in Figure A1 and Figure A2 below:

From the results, it can be seen that the PS points obtained from the PS−InSAR processing are too sparse, and there are basically no completely overlapping points in the two datasets, which makes it impossible to realize the fusion of multi-orbital time-series InSAR deformation. Therefore, for the Baige landslide, the PS−InSAR results can only be used to assist in recognizing the landslide trailing edge deformation area and cannot be used as the main basis for the division of landslide deformation stage or the division of landslide area.

## Appendix B

Pearson’s correlation coefficients were calculated according to the following Equation (A1):

(A1) $$\rho_{DR}=\frac{\sum_{i=1}^{n}\left(R_{i}-\bar{R})(D_{i}-\bar{D}\right)}{\sqrt{\sum_{i=1}^{n}{\left(R_{i}-\bar{R}\right)}^{2}}\sqrt{\sum_{i=1}^{n}{\left(D_{i}-\bar{D}\right)}^{2}}}$$

Among them, $\rho_{DR}$ represents the Pearson correlation coefficient between the deformation and the precipitation, $R_{i}$ is the precipitation in the ith month, $\bar{R}$ is the average precipitation from March to December, $D_{i}$ is the deformation in the ith month, and $\bar{D}$ is the average deformation from March to December.

Figure A3 below shows the stratigraphic lithology and topographic map around the Baige landslide:
